# Lysol and Kreolin Poisoning

**Published:** 1910-03-05

**Authors:** 


### SPECIAL ARTICLES.

### LYSOL AND KREOLIN POISONING.

The local and external effects of lysol in producing dermatitis of the fingers, which may be exceedingly troublesome to cure, are well known. Less familiar are the symptoms of poisoning by lysol taken internally, either by accident or with suicidal intent. Nevertheless it is very cheap and it is relatively easy to purchase, so that an increasing number of cases of poisoning by it are being recorded. Fortunately it is the exception for it to prove fatal, and the recovery of patients who may first be seen comatose and apparently moribund after taking lysol by the mouth is perhaps one of the most striking features of a large proportion of the cases. Nearly always there is a certain amount of damage to the tongue, the mouth, the fauces, the tonsils, and the pharynx, whilst now and then even the epiglottis and the larynx become acutely inflamed. The œsophagus seldom suffers and only in four out of seventeen consecutive cases recorded by Scharpff was there any definite evidence of a toxic effect upon the stomach or intestines. In one of the four cases there was vomiting which extended over several weeks, associated with abdominal pain and with melæna, indicative of actual lesions in the bowel; in another case the motions contained blood for a fortnight after the poison was swallowed, and in both the other cases there was similar damage to the intestines.

Just as in the case of carbolic acid, so in that of lysol, one never sees any very deep destruction of the mucous membrane, for the underlying tissue becomes immediately protected from further action of the poison by the rapid production of a white eschar composed of coagulated cell proteid and debris. The cure of the lesion, therefore, takes place very quickly as a rule and without the formation of an ulcer; there is irritation and inflammation, but only in one out of the seventeen cases mentioned above was there any indication of actual ulceration of the bowel. Another favourable circumstance is the relatively slow solubility of lysol and of the kresol contained in it, so that its resorption through the mucous membrane is necessarily delayed. It follows from this that there are never such large toxic quantities of it in the circulation at any one time as there may be in the case of more soluble poisons. It is all the more surprising, therefore, that the symptoms come on so rapidly. All phenol compounds seem to have the property of attacking the nervous system with great rapidity in the higher animals. Unconsciousness or coma supervenes very rapidly, and may last for twentyfour hours or longer without necessarily proving fatal. Smaller doses cause only headache and giddiness. Convulsions or muscular twitchings are not seen. The poison is apt to produce an extreme degree of cloudy swelling of the liver. Irritation of the kidneys is evidenced by a moderate degree of albuminuria with small numbers of fatty casts, leucocytes and epithelial cells, most of which disappear about the second week. A definite acute nephritis of serious degree does not occur in any but quite exceptional cases. Again and again, however, acetone is to be found in the urine, with or without diacetic acid at the same time. This is doubtless nothing more than a variety of inanition acetonuria, although it may be in part also due to a toxic proteid katabolism such as has been recorded in the case of other poisons. Reducing substances do not appear in the urine. Phenol is not excreted by the kidneys in the free state, but in combination with sulphuric acid, so that before it will give the ordinary phenol tests it has to be warmed or boiled with hydrochloric acid. There is a striking fact in most cases that the phenol persists in the urine for a considerable time, even up to nearly a fortnight after the poison has been taken. The body temperature of the patients seldom sinks very far below normal. Marked hypothermia (96° F. and 95.6° F.) was noted in only two out of the seventeen cases. A young woman, aged 27, about midday swallowed some 50 c.c. of undiluted lysol; she lost consciousness at once, and did not regain it until her stomach had been washed out and she had received infusion of saline solution. She was a strong sturdy woman. By next day she was troubled with hawking from her inflamed throat, and she brought up an abundance of greenish mucopurulent sputum. She complained of pain in her throat and also behind her sternum and in the chest and abdomen over the stomach region. Whitish eschars could be seen upon the hard palate, upon the left posterior pillar of the fauces, on the left tonsil, and upon the back of the pharynx. Examination on the third day showed an eschar extending down the pharynx; on the end of the epiglottis, over both arytenoid cartilages, and on the left aryteno epiglottidean fold there were similar greyish-white eschars. The lung signs were normal except for an occasional rhonchus. The rate of the heart beat was about 150 per minute, but otherwise the cardiac physical signs were natural. There was tenderness in the epigastrium and a large mass of fæces could be felt in the intestines.

The urine, which was already moderately dark when passed, became still darker when it stood exposed to the air; but it gave no ferric chloride reaction and it contained no sugar. There was a slight turbidity on testing for albumin. In the centrifugalised deposit there were large numbers of cubical and of tailed epithelial cells but no tube casts. The respiration rate was about 50 and the temperature 102.5° F. On the following day the temperature was still up to 100.7° F., and there were the symptoms and signs of broncho-pneumonia in the left lung. The amount of sputum on the second day amounted to about 400 c.c., and even on the eighth day to 10 c.c. The temperature reached normal by the eighth day, and from that time the lung condition slowly but steadily improved, though abnormal physical signs could still be detected to some extent three weeks later. By the third week the mouth, pharynx, and larynx were free from eschars, but the mucous membrane was still much reddened where the eschars had been; nevertheless there was still a good deal of pain in the throat and in the stomach for some weeks after this. In this patient no blood could be detected in the fæces.

Seeing that kreolin is so similar a substance to lysol in certain respects, it may be of interest to describe a case of poisoning by it, also recorded by Scharpff. A workman, aged 28, was brought to hospital one evening about 8.30 P.M., about some half-hour after he had drunk 250 c.c. of pure kreolin when he was in a temper about a quarrel at home. His last meal had been at 4 o'clock. He had immediately suffered from acute abdominal pain and had forthwith lost consciousness. His stomach was washed out at 9 P.M. He was a big powerful man. Neither the mouth nor pharynx showed any eschars, the only point to note about the mouth being slight reddening of the palate. The heart and lungs were normal. The epigastrium was not particularly tender to pressure. The urine was of a dark brown colour and contained a slight amount of proteid, but no abnormal cell elements, no sugar, no acetone, and no urobilin. On distilling it pure kreolin was given off. The patient's temperature was 98° F., and the pulse rate about 76. The trace of proteid in his urine disappeared on the fourth day, and from the fifth day onwards the urine contained no abnormal constituents, and it was of a normal colour. The patient was discharged quite well at the end of a week.

Dinter records kreolin poisoning in three lunatic women in 1889. In one of them coma developed rapidly, and collapse with a cold skin and feeble pulse were other symptoms. In the other two there were severe erosions of the tongue; unlike the case recorded above, there were more or less severe gastro-intestinal symptoms with vomiting and the passage of thin greenish fluid per rectum smelling of kreolin. The first two cases had the hypothermia of collapse down to 94° F., whilst the third had a tendency to fever. In all three recovery was rapid and complete. Ackeran records a case of a workman, aged 30, who had drunk 200 c.c. of kreolin. He, like the others, became comatose. From the second to the ninth day he suffered from cramps in his upper extremities and paræsthesia in the region of the radial nerve. He also showed acute nephritis, jaundice, and enlargement of his liver and spleen. His urine continued to contain kreolin until after the ninth day, but ultimately recovery was complete.

Piller records the case of a woman aged 60 who had drunk 75 c.c. of kreolin. She became unconscious, vomited, and passed diarrhœic stools containing much kreolin. The urine smelled of kreolin and darkened on standing. It contained traces of proteid, red and white corpuscles, and also kidney and vesical epithelial cells. The patient was practically well by the eighth day.

It will be seen, therefore, that almost without exception phenol derivatives when taken in poisonous doses lead to a rapid interference with the central nervous system, as evidenced by loss of consciousness, which may readily deepen into coma.

The corrosive action of kreolin seems to be very slight, though now and then it may cause some excoriation of the tongue. As regards the glandular organs, one nearly always finds some evidence of irritation of the kidney, but whether this is slight, as in most cases, or severe, as in a few, it nearly always quickly resolves. The gastro-intestinal system suffers to a very variable degree; if it is affected at all as a rule there is considerable vomiting and profuse diarrhœa, with the odour of kreolin, but occasionally instead of diarrhœa and vomiting there may be extreme abdominal pain.

